# Why Did Bluetongue Spread the Way It Did? Environmental Factors Influencing the Velocity of Bluetongue Virus Serotype 8 Epizootic Wave in France

**DOI:** 10.1371/journal.pone.0043360

**Published:** 2012-08-15

**Authors:** Maryline Pioz, Hélène Guis, Laurent Crespin, Emilie Gay, Didier Calavas, Benoît Durand, David Abrial, Christian Ducrot

**Affiliations:** 1 Institut National de la Recherche Agronomique, UR346 d'Epidémiologie Animale, Paris, France; 2 CIRAD, UMR CMAEE, Montpellier, France; 3 Laboratoire de Biométrie et Biologie Evolutive, Université de Lyon, Lyon; Université Lyon 1; CNRS, UMR5558, Villeurbanne, France; 4 French Agency for Food, Environnemental and Occupational Health Safety, Lyon, France; 5 French Agency for Food, Environnemental and Occupational Health Safety, Maisons-Alfort, France; University of Liverpool, United Kingdom

## Abstract

Understanding where and how fast an infectious disease will spread during an epidemic is critical for its control. However, the task is a challenging one as numerous factors may interact and drive the spread of a disease, specifically when vector-borne diseases are involved. We advocate the use of simultaneous autoregressive models to identify environmental features that significantly impact the velocity of disease spread. We illustrate this approach by exploring several environmental factors influencing the velocity of bluetongue (BT) spread in France during the 2007–2008 epizootic wave to determine which ones were the most important drivers. We used velocities of BT spread estimated in 4,495 municipalities and tested sixteen covariates defining five thematic groups of related variables: elevation, meteorological-related variables, landscape-related variables, host availability, and vaccination. We found that ecological factors associated with vector abundance and activity (elevation and meteorological-related variables), as well as with host availability, were important drivers of the spread of the disease. Specifically, the disease spread more slowly in areas with high elevation and when heavy rainfall associated with extreme temperature events occurred one or two months prior to the first clinical case. Moreover, the density of dairy cattle was correlated negatively with the velocity of BT spread. These findings add substantially to our understanding of BT spread in a temperate climate. Finally, the approach presented in this paper can be used with other infectious diseases, and provides a powerful tool to identify environmental features driving the velocity of disease spread.

## Introduction

Predicting the course and geographic spread of an infectious disease is critical for its control [1]. It enables health agencies to respond to a disease wave by implementing preventive measures to limit contagion, *i.e.*, through vaccination or animal movement restrictions [2]–[8]. Knowledge of the main factors influencing the speed of spread is needed to plan such interventions, which can reduce greatly the consequences of the disease. Numerous factors drive the spread of a vector-borne disease such as bluetongue (BT) [9]. These factors can be abiotic (suitability of the environment for disease transmission), vector-related (abundance, activity), or host-related (host availability, immunity). The major Bluetongue Virus Serotype 8 (BTV-8) outbreak experienced by northern Europe in 2006–2008 offers an opportunity to identify the major factors influencing the velocity of BT spread in a temperate climate context.

Bluetongue Virus (BTV) is transmitted to ruminants by biting midges of the genus *Culicoides.* In southern Europe, Africa and Asia the major BT vector is *Culicoides imicola.* In northern Europe, where *C. imicola* is absent, species of the Obsoletus Group, *i.e., Culicoides obsoletus*, *Culicoides scoticus*, *Culicoides dewulfi* and *Culicoides chiopterus* [10], have been identified as major BT vectors [11]–[15]. Unfortunately the biology of *Culicoides*, specifically of these indigenous Palaearctic vector species, is poorly understood [16], [17]. In particular, there is scant information about the life history traits of midges affecting the spread of BT [18], [19]. After the introduction of BTV-8 in the Netherlands in the summer of 2006, BT spread rapidly across northwest Europe; by the end of 2008, more than 86,000 holdings in 14 countries were reported to be affected by BTV-8 [17]. France was impacted heavily with more than 43,000 holdings affected over the 2007–2008 period. The epidemic wave progressed from north-eastern to south-western France at an average velocity of 5.6 km/day [20]. However, the disease did not spread at the same velocity throughout the country. The infection progressed more rapidly in some geographic areas that seemed to act as corridors facilitating the spread of the disease (see Figure 6 in Pioz et al. 2011 and online Appendix video in [21] www.cdc.gov/EID/content/16/12/1861-appV.htm). On the other hand, some areas were characterized by a slow progression of the infection. In the absence of detailed knowledge of *Culicoides* biology, determining the ecological characteristics of areas associated with rapid and slow BT spread is crucial to enhancing knowledge on the epidemiology of BT.

Since the BTV-8 outbreak in northern Europe, much research has been devoted to modelling BTV epidemics and developing models that assess the risk of BT or the probability of vector presence based on a set of eco-climatic factors [5], [18], [21]–[31]. Statistical models have explored the relationship between a range of predictor variables and the occurrence of BT cases, but none have investigated the relationship between predictor variables and the velocity of spread of the infection. The identification of the most important factors influencing the velocity at which the travelling wave of BT diffused across large areas would complement other modelling efforts, such as transmission models for BTV or predictive models of BT spread. However, as environmental and ecological data generally show spatial autocorrelation, we advocate the use of Simultaneous Autoregressive (SAR) models [32], which account for spatial autocorrelation, to investigate the environmental drivers influencing the velocity of disease spread. This approach, which is applicable to any infectious disease, renders it possible to disentangle the main environmental features that influence the velocity of disease spread. A variety of models can be used to study the spatial spread of disease. Individually-based models, which account for the spatial interaction between hosts distributed on a landscape, and integro-differential equations, which use a spatial transmission kernel, were both used to model the foot-and-mouth disease spread in the United Kingdom in 2001 [33], [34]. Reaction-diffusion models, which assume local transmission and rely on spatial diffusion of hosts to spread the infection, were used to model the spread of rabies in red fox *Vulpes vulpes* [35]. Metapopulation models were used to investigate the spread of phocine distemper virus in harbour seal *Phoca vitulina* [36] or the spread of rabies in raccoon *Procion lotor* [37]. These modeling approaches are sophisticated and require biological knowledge on the disease transmission as well as complex parameterization. Instead, our modelling approach is simpler in the sense that transmission is not explicitly modelled, but it still allowed us identifying the environmental features that influence the velocity of BT spread. Such knowledge would facilitate the adoption of relevant containment strategies. Indeed, as landscape features may serve as barriers or gateways to the spread of diseases, understanding the way they impact disease spread could lead to improved control strategies. For example, vaccination is an effective tool to control BT spread [5], [38], but the timing of vaccination of susceptible populations is crucial [38], [39]. Vaccination failed to control and stop BT spread in France in summer 2008 because it was implemented late regarding the disease wavefront. Susceptible animals need to be vaccinated ahead of the disease wavefront, and early enough to achieve full protection. Hence, identifying environmental conditions that slow down or speed up the disease spread would help health agencies to foresee where and when the wavefront of the disease would reach each area, and consequently adapt the vaccination strategy. The objective of this study was therefore to explore the environmental factors that impacted the velocity of BT spread in France during the 2007–2008 epizootic wave.

## Materials and Methods

### Estimates of bluetongue velocity

We used the velocities of BTV-8 spread across France in 2007–2008 that were estimated from a Trend-Surface Analysis model combined with a spatial error Simultaneous Autoregressive model (trend SAR_err_ model) to account for spatial autocorrelation [20]. In the Pioz study [20], velocities were estimated for 10,994 French municipalities, *i.e.*, the smallest French administrative subdivision, based on the date of the first BTV-8 clinical case reported in the municipality. Given that the aim of the present study was to identify the major environmental factors influencing the velocity of BT spread, we had to use the most accurate estimations of velocity of BT spread. With regard to the 10,994 municipalities, we could assume that the estimated velocity of BTV-8 spread was close to the real velocity at which the infection spread in the area if the predicted date of the first case was close to the observed one. The difference between the predicted and observed date of the first clinical case ranged between −221 and +162 days (mean  = 0 day, standard deviation  = 32.3 days). The method used for the estimation of the velocity, *i.e.*, the trend surface analysis, is known to be sensitive to edge effect, so that interpretation at the edges of the study area should be made with caution. Given that most municipalities with a large error regarding the predicted date were situated at the edge of the study area, edge effect was indeed probably the main reason for the incorrect prediction. In the present study, we consequently restricted the dataset to the 5,993 municipalities for which the difference between the observed date of the first clinical case and the date predicted by the model of Pioz et al. [20] was less than 16 days. However, we checked that the range and characteristics of the environmental factors in the restricted dataset remained similar to that one in the full dataset. The response variable is the velocity of BTV-8 spread in 5,993 French municipalities in 2007–2008.

### Ecological variables

Sixteen covariates defining five thematic groups of related variables were tested (Table 1). Host availability, vaccination, elevation and landscape-related variables were obtained at the municipality-level. Meteorological-related variables were obtained on an 8×8 km square grid through the SAFRAN database supplied by Météo-France [40]. Restrictions on animal movements in France following Directive 2000/75/EC and Commission Regulation No 1266/2007 prevented all movement of infected farm animals from restricted zones (70-km radius around the contaminated farms) to non-restricted zones. As we studied the velocity of spread of the BT front-wave, we focused on the BT spread over newly-contaminated areas, and considered that the movements of infected farm animals explaining the velocity of spread was negligible due to the ban (see [20] for a discussion on the negligible effect of movements of infected animals).

**Table 1:** Description of the 16 environmental explanatory variables used to model the velocity of BTV-8 spread in France in 2007–2008.

Covariate	Definition	Resolution	Type (continuous *vs* categorical)	Number of classes	Classes							
					a		b		c		d	
					range	n	range	n	range	n	range	n
Elevation	Average elevation of the municipality(in meters above sea level)	municipality	categorical	4	2–127	1499	127–221	1498	222–358	1498	358–1915	1498
Rain_lag1	Total monthly rainfall the month prior to the 1st clinical case of BTV-8 (in mm)	grid 8×8 km	categorical	4	13.1–42.8	1499	42.8–57.5	1498	57.5–84.5	1498	84.5–324	1498
Rain_lag2	Total monthly rainfall two months priorto the 1st clinical case of BTV-8 (in mm)	grid 8×8 km	categorical	4	13.3–50.9	1499	50.9–75.9	1498	75.9–106	1498	106–323.9	1498
Tmax_lag1	Monthly average of the maximal daily temperature the month prior the 1st clinical case of BTV-8 (in °C)	grid 8×8 km	categorical	4	4.5–18.97	1499	18.97–21.35	1498	21.35–23.11	1498	23.11–25.75	1498
Tmax_lag2	Monthly average of the maximal daily temperature two months prior the 1st clinical case of BTV-8 (in °C)	grid 8×8 km	categorical	4	6.89–19.77	1499	19.77–20.90	1498	20.90–22.15	1498	22.15–28.45	1498
DensBeef_Cattle	Density of beef cattle older than 2 months in 2007 (number per km^2^)	municipality	categorical	4	0–4.9	1076	5–19.9	1510	20–39.9	1497	40–497	1920
DensDairy_Cattle	Density of dairy cattle older than 2 months in 2007 (number per km^2^)	municipality	categorical	4	0–4.9	1878	5–19.9	1582	20–39.9	1106	40–353	1427
DensSheep	Density of sheep in 2000(number per km^2^)	municipality	categorical	4	0	3270	0.03–3.9	946	4–19.9	884	20–770	893
vaccination	Percent of vaccines acquired in the department 2 months prior to the 1st clinical case of BTV-8 in the municipality	municipality	categorical	3	0	3611	1–29	1143	30–100	1239		
Arable-pasture	Edge density between arable land and pastures (in m/ha)	municipality	categorical	4	0	1863	0.001–2.289	1377	2.290–6.200	1377	6.201–29.770	1376
Arable-forest	Edge density between arable land and deciduous and mixed forests (in m/ha)	municipality	categorical	4	0	2464	0.003–0.895	1176	0.896–2.760	1176	2.761–29.080	1177
Forest-pasture	Edge density between deciduous and mixed forests and pastures (in m/ha)	municipality	categorical	4	0	1353	0.001–1.414	1547	1.415–3.937	1547	3.938–26.440	1546
SIDI	Simpson's Diversity Index	municipality	continuous		0–0.84	5993						
p_arable	Percentage of arable land in the municipality (in %)	municipality	continuous		0–100	5993						
p_pasture	Percentage of pastures in the municipality (in %)	municipality	continuous		0–100	5993						
p_forest	Percentage of deciduous and mixed forests in the municipality (in %)	municipality	continuous		0–86	5993						

For categorical variables number of classes, range and number of municipalities in each class are presented, for continuous variables range is presented. N = 5,993 municipalities.

#### Host availability variables

Density of beef cattle and dairy cattle in September 2007 was obtained from the French National Cattle Register (BDNI). Because of maternal immunity, we only considered cattle over 2 months old to reflect the population size of susceptible hosts. Sheep density was obtained from the exhaustive census realized throughout France by the French Ministry of Agriculture in 2000, the most recent estimates we could obtain. Overall, the global number of sheep decreased between 2000 and 2007 in France. However, the relative differences of sheep density across geographical areas were similar between 2000 and 2007. We therefore assumed that the 2000 census approximately reflected the relative number of sheep per municipality in 2007. Densities were expressed in number of animals per km^2^. A change in the host density is expected to modify the velocity of BT spread as it will change the probability of transmission from vector to host. Furthermore, different host species may change the velocity in different ways because of differences in susceptibility, capability to transmit the disease and management practices. Consequently, the density of sheep, beef cattle and dairy cattle were not expected to have the same influence on the velocity of BT spread.

#### Vaccination

Precise data on BTV-8 vaccination were not available so we defined a proxy to estimate the level of immunity in the municipality when the first clinical case was detected. Hypothesizing that there was a delay of two to three weeks between the arrival of vaccine doses in a department and their use in the field by veterinarians, we used the percentage of vaccine doses acquired in a department two months prior to the date of the first BTV-8 clinical case in a municipality as a proxy of the relative immunity in a municipality (see Text S1 for details). We expected the slowest velocities of BT spread in areas with a high relative level of immunity.

#### Elevation

Average elevation of each municipality measured in meters above sea level was extracted from the database GEOFLA® 2002, edition 6, of the Institut Géographique National (IGN). Average elevation is calculated by IGN from elevation obtained at a resolution of 50 meters. Elevation may influence the velocity of BT spread through its effect on vector abundance and *Culicoides* species composition.

#### Landscape-related variables

Municipality-specific land cover data were extracted from the 2006 version of the CORINE (Coordination de l'Information sur l'Environnement) Land Cover database (CLC), provided by the European Environment Agency at a resolution of 100 meters [41]. Landscape components may be important in defining favourable environments for BT [28]. Durand et al. [21] identified three landscape associated with BTV-8 seropositivity: forests, pastures and arable land. Furthermore, the breeding habitat of *Culicoides obsoletus* sensu stricto (and possibly *C. scoticus*), the major vector involved in BTV-8 transmission in northern Europe, is the leaf litter of deciduous trees such as the common beech *Fagus sylvatica* [42], [43]. We consequently discarded coniferous forests from the analysis and extracted from the 44 CLC landscape classes the proportion of the municipality area covered by deciduous and mixed forests, arable land and pastures ( Table S1). For each pair of these classes, we calculated the edge density, which is the length of the edges between two classes divided by the municipality area (3 variables in m/hectare). Finally, we calculated an index of diversity to characterize the landscape diversity in each municipality. Simpson's Diversity Index (SIDI) is a popular diversity measure in community ecology [44]. It has an intuitive interpretation as it represents the probability that any 2 randomly selected pixels would be different patch types. SIDI  = 0 when the landscape contains only 1 patch, and approaches 1 as the number of different patch types increases, and the proportional distribution of area among patch types becomes more equitable. Overall, seven landscape-related variables were tested. Landscape-related variables may change the velocity of BT spread through their influence on *Culicoides* abundance and species diversity as well as on the probability of contact between hosts and vectors.

#### Meteorological-related variables

Both the BTV transmission cycle and the lifecycle of its *Culicoides* vectors are affected by temperature and humidity [18], [45]. Short term meteorological conditions rapidly can change the age structure and density of *Culicoides* populations, thus modifying the number of midges that can transmit the virus, and consequently the rate of disease transmission [46]. Meteorological conditions also affect the daily flight activity of *Culicoides* vectors [47]. We thus were interested in capturing the meteorological conditions around the period at which the first animal became infected in a municipality. To account for the uncertainty of the date of the infectious bite, we investigated the effect of temperature and rainfall up to two months prior to the date of the first clinical case reported in each municipality (see Text S1 for details). We consequently considered the monthly average of maximal daily temperatures and monthly total rainfall one month and two months before the first case of BTV-8. These meteorological covariates are referred to as one month-lag and two month-lag, respectively.

To identify non-monotonic and nonlinear responses and determine whether the covariates should be considered as continuous or categorical, we examined the linearity of the relation between each continuous covariate and the response variable. Based on biological relevance, each continuous covariate was discretized: it was divided into four classes of approximately similar size, with the exception of the vaccination covariate which was divided into three classes. The response variable, *i.e.*, the velocity of BT spread, was then plotted against each discretized categorical variable, and we visually examined each graph to detect nonlinear variations. If the variation was approximately linear between each class, we used the continuous covariate as a candidate variable in the model; otherwise, we used the categorical covariate. Finally, we obtained 4 continuous and 12 categorical candidate variables. Their characteristics are summarized in Table 1. To prevent multicollinearity, we avoided including highly correlated covariates simultaneously in a model. Before discretization, correlations among all the candidate variables were assessed using Spearman's rank correlation ρ because the covariates were not normally distributed. All of the ρ were lower than |0.64|. The covariates were not highly correlated and could be included simultaneously in a model. Only plausible two-way interactions were considered, *i.e.*, the interaction between temperature and rainfall at the same lag time, and the interaction between sheep density and each of the cattle densities (dairy and beef). Overall, 16 candidate covariates along with 4 plausible biological interactions between candidate variables were tested.

### Statistical analysis

#### Model selection

**Figure 1:**
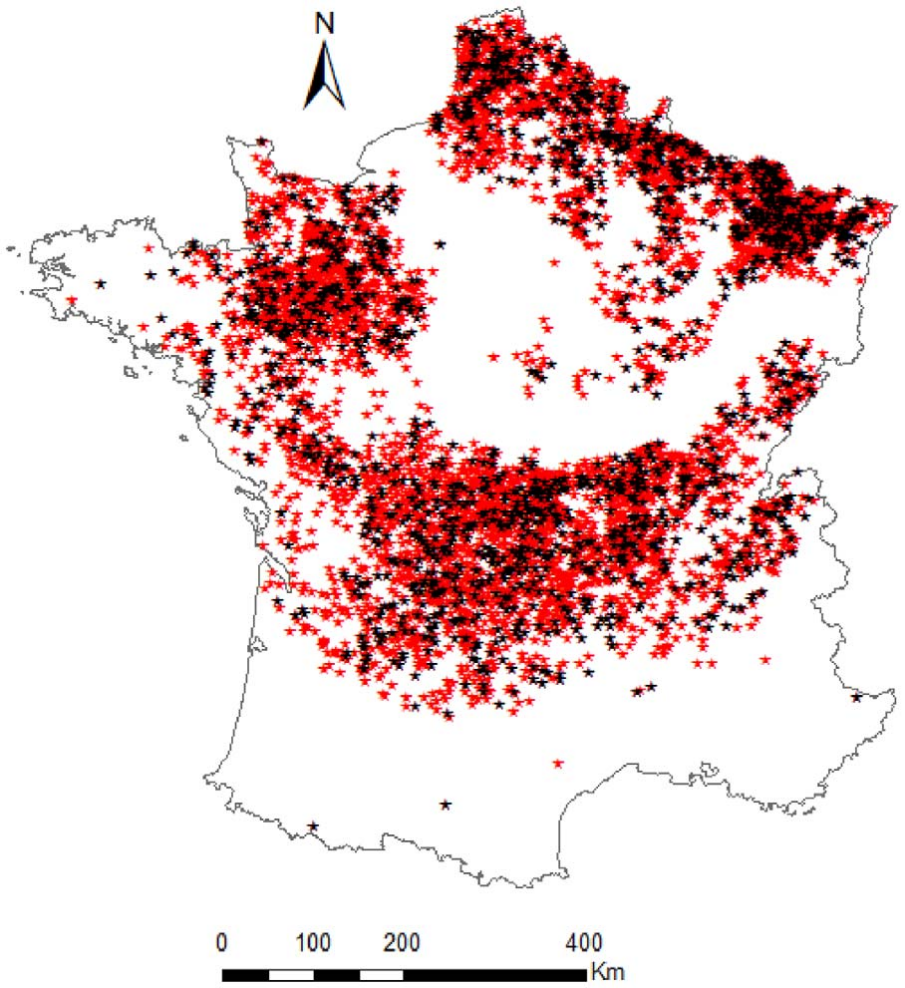
Spatial distribution of the 5,993 French municipalities. The 4,495 municipalities of the building dataset and the 1,498 municipalities of the validation dataset are represented by red stars and black stars, respectively.

**Figure 2:**
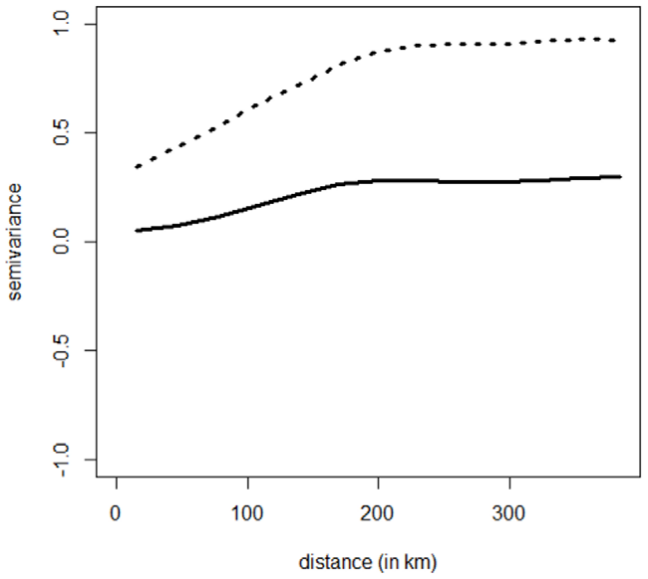
Semi-variogram of the OLS model (dashed line) and the SAR_err_ model (solid line).

The original dataset of 5,993 municipalities was split randomly into a “model building” dataset (75% of the data) and a “validation” dataset (25% of the data), representing 4,495 municipalities and 1,498 municipalities, respectively (Figure 1). We initially applied standard linear regression methods based on ordinary least squares (OLS) on the model building dataset. However, a strong spatial autocorrelation of the residuals indicated that the assumption of independent errors was violated. We consequently extended the model to account for the spatial dependency and used a simultaneous autoregressive model (SAR) [48]. All of the statistical analyses were realized using R software v2.13.1 [49]: spatial models were fitted using the package spdep [50] and geoR [51], respectively. We determined the type of spatial dependence model to use (spatial lag or spatial error) based upon the largest value of the robust Lagrange Multiplier indicators [1], [52], which were obtained by using the lm.LMtests function of the spdep package [50]. Based on the results of the Lagrange Multiplier tests (Table S2), we applied the spatial error form of the SAR model (hereafter referred to as spatial error model or SAR_err_ model) [48], [53]. Details on SAR_err_ models are provided in Text S2. Based on the semi-variogram of the OLS residuals (Figure 2), residuals were autocorrelated until 200 km. We consequently considered a 200 km radius spatial neighbourhood. We used a neighbourhood matrix with a variance stabilizing ‘S’ coding style [54], and with the weights set to be proportional to the inverse distance between the centroids of the municipalities (see Text S2 for details).

We used backward model selection based on AIC to select the best model based on both model fit and model complexity [55]. As recommended by Burnham and Anderson [55], we considered that two nested models differing by less than 2 AIC points received identical support from the data. In such a situation, the model with fewer parameters was preferred. However, the large number of candidate variables meant that a huge number of models could be considered. Thus, for practical reasons, we used a hierarchical approach that allows us to isolate independently for each of the three thematic sets of variables (host-, meteorological- and landscape-related covariates) a combination of variables best fitting the data. These three combinations were then added in a global model from which the final backward selection was made. In order to evaluate the difference in direction, magnitude and significance of coefficients before and after correction for spatial autocorrelation, we also presented the result obtained from the best OLS model. Because the assumption of independence is violated in the presence of autocorrelation, the traditional R^2^ measure of fit is not applicable to the spatial autoregressive model. In the literature several pseudo-R^2^ have been defined and used for spatial models. One of the most popular and frequently used measure is the coefficient of determination, *i.e.*, the squared Pearson correlation r between predicted and observed values [32], [56]. The squared Pearson's r provides a measure of goodness of fit of the model. We also calculated the Root Mean Squared Error (RMSE). RMSE is one of the most widely used measures of model uncertainty and quantifies the precision of the prediction error [57]. RMSE is a good measure of how accurately the model predicts the response, with lower values of RMSE indicating a better fit.

#### Model validation

We used the 1,498 municipalities of the validation dataset to evaluate the predictive power of the model. Although one can not expect an explanatory model to be optimal in terms of predictive power, it should show some degree of accuracy [58]. As for the training dataset, we calculated the squared Pearson correlation between the observed and predicted velocities as well as the Root Mean Squared Error (RMSE).

#### Model interpretation

To assess the relative importance of environmental variables in the selected SAR_err_ model, we evaluated the contribution of each covariate to model fit with a likelihood ratio (LR) test for nested models [48], [59]:
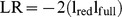
where LR is the likelihood ratio test statistic, and l_red_ and l_full_ are the log-likelihoods of the reduced and full models, respectively. The full model is the SAR_err_ model and the reduced model contains all but one of the variables of the full model. The contribution of the omitted variable is thus evaluated, larger LR values indicating a greater contribution to model fit. LR values for the best OLS model also were calculated to compare the relative importance of environmental variables in the OLS and SAR_err_ models.

## Results

### Model selection

Hierarchical model selection conducted within each of the three sets of models, *i.e.*, meteorological-, landscape- and host-related covariates, as well as the final model selection, conducted after combining the remaining predictors, are detailed in Table S3. The final SAR_err_ model contained thirteen covariates and three interactions (Table 2). The edge density between arable lands and pastures, the percentage of area of deciduous and mixed forests in municipality, and the density of beef cattle were dropped from the final model. The fit of the best model was satisfactory (squared Pearson's r = 0.93, RMSE  = 0.52 km/day with an average velocity of BT spread equaling 5.5 km/day). In contrast to the OLS model, inspection of the residuals from the final SAR_err_ model showed low spatial autocorrelation (Figure 2). Residual autocorrelation was still present, but greatly reduced in comparison to the OLS regression, underscoring the greater fit of the data by the spatial model. In comparison, the OLS model with the lowest AIC had a R^2^ = 0.57, a greater amount of autocorrelation (Figure 2), and showed some differences in the selected covariates (Table S4).

**Table 2:** Parameter estimates, 95% Confidence Interval (CI) and p-values of the selected final SAR_err_ model for the 4,495 French municipalities.

covariates	classe	coefficient	95%CI	p-value
intercept		4.93	4.752; 5.117	<0.001
elevation	b	0.07	0.020; 0.126	0.01
	c	−0.26	−0.332; −0.185	<0.001
	d	−0.62	−0.716; −0.531	<0.001
DensDairy_Cattle	b	−0.07	−0.132; −0.013	0.02
	c	−0.16	−0.223; −0.089	<0.001
	d	−0.38	−0.448; −0.309	<0.001
DensSheep	b	−0.09	−0.168; 0.009	0.08
	c	−0.07	−0.152; 0.011	0.09
	d	−0.07	−0.147; 0.014	0.11
vaccination	b	−0.15	−0.203; −0.096	<0.001
	c	−0.22	−0.275; −0.164	<0.001
Rain_lag1	b	0.04	−0.046; 0.133	0.34
	c	−0.05	−0.146; 0.050	0.34
	d	−0.19	−0.291; −0.090	<0.001
Tmax_lag1	b	0.07	−0.031; 0.174	0.17
	c	0.41	0.294; 0.526	<0.001
	d	0.34	0.223; 0.459	<0.001
Rain_lag2	b	0.00^a^	−0.107; 0.099	0.94
	c	0.15	0.043; 0.258	0.01
	d	0.12	0.014; 0.220	0.03
Tmax_lag2	b	−0.08	−0.186; 0.023	0.13
	c	−0.01	−0.122; 0.106	0.89
	d	0.01	−0.104; 0.124	0.86
SIDI		−0.17	−0.329; −0.020	0.03
p_arable		0.00^b^	0.003; 0.005	<0.001
p_pasture		0.00 c	0.003; 0.005	<0.001
arable-forest	b	0.02	−0.027; 0.067	0.40
	c	0.04	−0.009; 0.092	0.11
	d	0.11	0.058; 0.172	<0.001
forest-pasture	b	0.03	−0.021; 0.081	0.24
	c	0.05	−0.003; 0.110	0.06
	d	0.09	0.025; 0.153	0.01
interactions				
1st term	2nd term			
DensDairy_Cattle b	DensSheep b	0.01	−0.106; 0.131	0.83
	DensSheep c	−0.03	−0.146; 0.090	0.64
	DensSheep d	0.03	−0.083; 0.145	0.60
DensDairy_Cattle c	DensSheep b	0.02	−0.113; 0.149	0.79
	DensSheep c	0.07	−0.061; 0.203	0.29
	DensSheep d	0.03	−0.112; 0.167	0.70
DensDairy_Cattle d	DensSheep b	0.23	0.097; 0.357	<0.001
	DensSheep c	0.34	0.207; 0.467	<0.001
	DensSheep d	0.29	0.140; 0.442	<0.001
Tmax_lag1 b	Rain_lag1 b	−0.06	−0.193; 0.077	0.40
	Rain_lag1 c	0.09	−0.046; 0.2227	0.20
	Rain_lag1 d	0.40	0.260; 0.538	<0.001
Tmax_lag1 c	Rain_lag1 b	−0.23	−0.357; −0.101	<0.001
	Rain_lag1 c	−0.16	−0.301; −0.025	0.02
	Rain_lag1 d	−0.13	−0.280; 0.018	0.09
Tmax_lag1 d	Rain_lag1 b	−0.19	−0.322; −0.055	0.01
	Rain_lag1 c	−0.14	−0.287; −0.002	0.05
	Rain_lag1 d	−0.22	−0.367; −0.074	<0.01
Tmax_lag2 b	Rain_lag2 b	0.17	0.035; 0.311	0.01
	Rain_lag2 c	0.14	−0.001; 0.282	0.05
	Rain_lag2 d	0.08	−0.057; 0.214	0.26
Tmax_lag2 c	Rain_lag2 b	0.17	0.021; 0.315	0.03
	Rain_lag2 c	−0.08	−0.225; 0.069	0.30
	Rain_lag2 d	−0.25	−0.406; −0.103	<0.01
Tmax_lag2 d	Rain_lag2 b	−0.08	−0.212; 0.053	0.24
	Rain_lag2 c	−0.36	−0.499; −0.202	<0.001
	Rain_lag2 d	−0.37	−0.541; −0.193	<0.001
lambda		0.89		

See Table 1 for description of covariates. SAR_err_ Simultaneous Autoregressive Error model.

a −0.0043.

b 0.0039.

c 0.0041.

### Model validation

Overall, the model performance in term of predictive power may be seen as relatively low (squared Pearson's r = 0.43, RMSE  = 1.42 km/day). However, this was expected as the model is an explanatory model, which is not devoted to making predictions.

### Model interpretation

**Figure 3:**
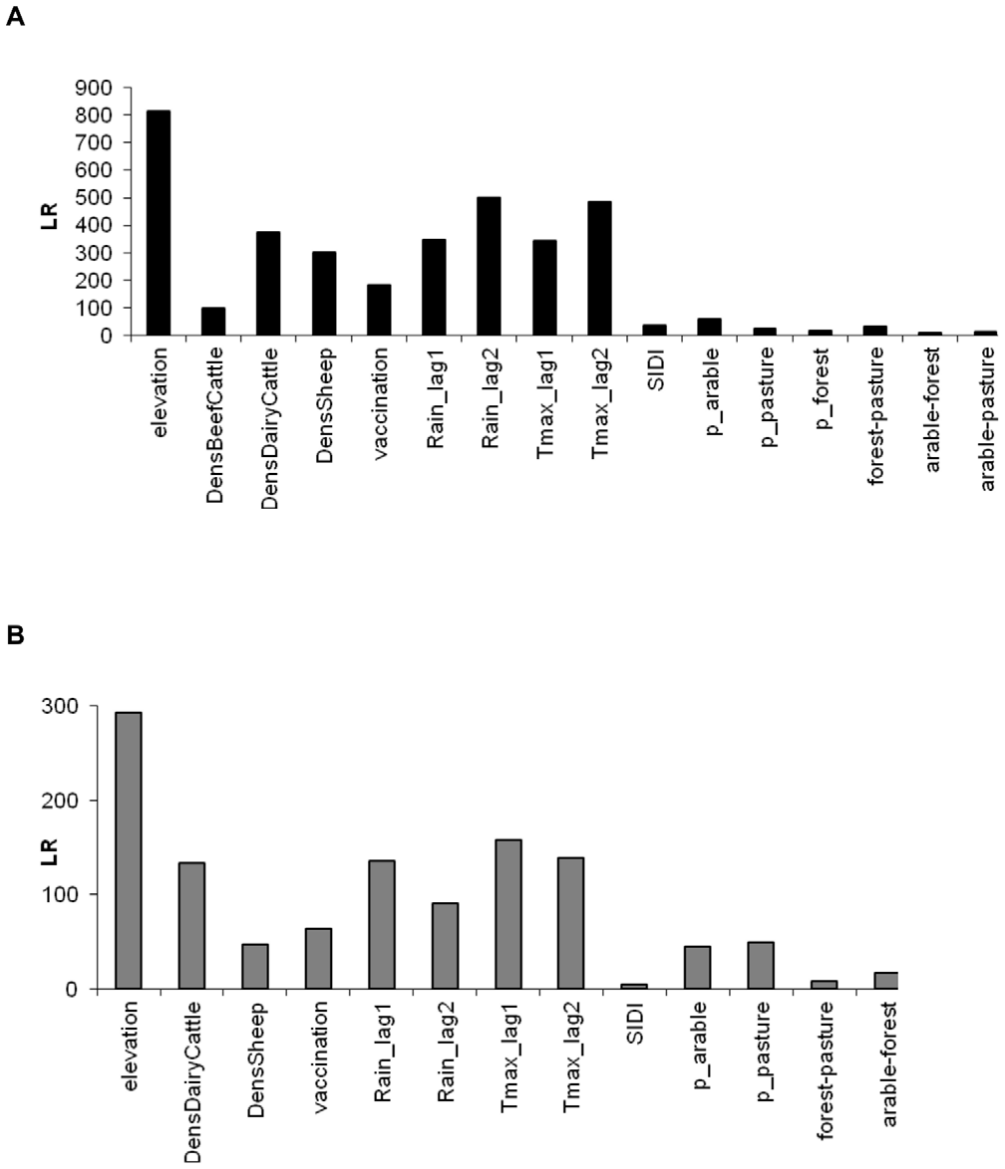
Likelihood ratio (LR) statistics for environmental variables in A) OLS and B) SAR_err_ models. Larger LR values indicate a greater contribution to model fit. P-values and parameter estimates of the OLS and SAR_err_ models are given in Tables S4 and 4, respectively. See Table 1 for descriptions of environmental variables.

The contribution of covariates to model fit was assessed through the comparison of the LR-values of each covariate (Figure 3). The comparison of LR-values from the final SAR_err_ model (Figure 3B) with those from the best OLS model (Figure 3A) shows that the effect of the covariates was reduced and more contrasted when autocorrelation was accounted for. Furthermore, the relative importance of covariates shifted across the OLS and SAR_err_ models. Regarding the SAR_err_ model, six out of thirteen covariates had a relative higher importance on influencing the velocity of spread: elevation, the four meteorological-related variables, and the density of dairy cattle. Four other variables, the index of vaccination in the municipality, the percentage of pastures, the density of sheep and the percentage of arable land also influenced the velocity of spread. The three remaining landscape-related variables only influenced the velocity of BT spread slightly.

**Figure 4:**
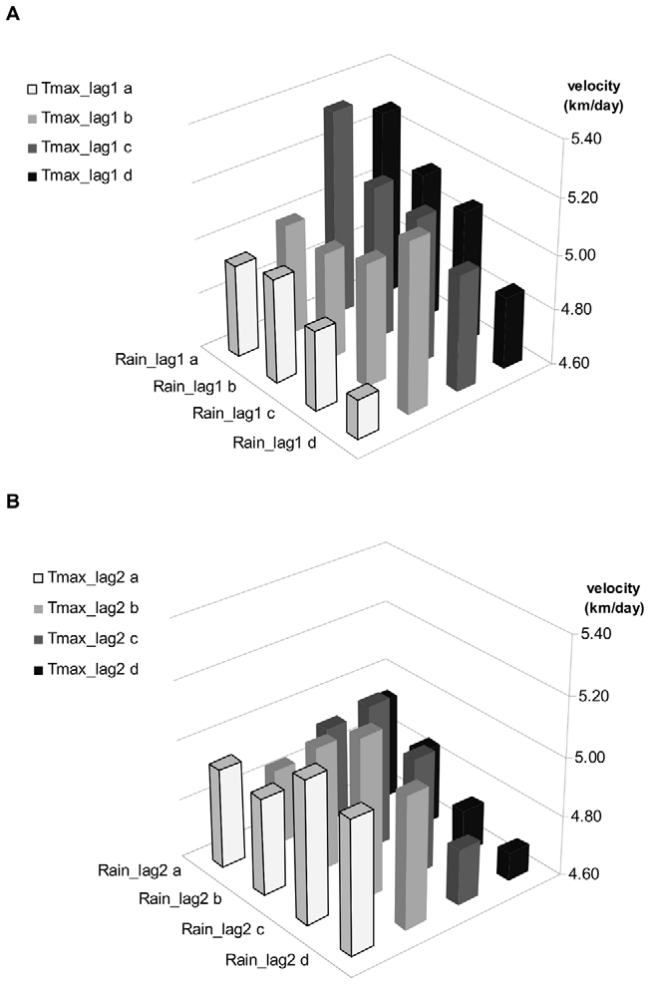
Effect of meteorological conditions on the velocity of BTV-8 spread in France in 2007–2008. The effect of the interaction between the monthly average of maximum daily temperatures and monthly total rainfall is represented through the value of the predicted velocity associated with each combination of temperature and rainfall at A) one month-lag, and B) two month-lag.

The effect of environmental covariates on velocity is reported holding all the other covariates constant, and for an average velocity of BT spread across the country of 4.9 km/day (Table 2). Regarding the ten most important covariates, the velocity of BT spread was negatively associated with elevation: velocity decreased by 0.26 km/day for elevation range between 222 and 358 m, and by 0.62 km/day for elevation >358 m. The effects of meteorological-related variables are presented in Figure 4 and Tables S5A and B. As rainfall changed the effect of temperature, the two covariates had to be interpreted together both at the one month-lag and two month-lag. Considering the effect of weather at the one month-lag, the highest velocities were observed when the monthly average of maximum daily temperature was between 21 and 26°C with minimal monthly rainfall (<43 mm). The lowest velocities were observed for maximal rainfall (>84.5 mm) associated with the lowest and highest classes of temperatures (<18.9 or >23.1°C). Considering the effect of weather at the two month-lag, the highest velocities were observed when the monthly average of maximum daily temperature was around 20°C with medium values of rainfall (between 75 and 106 mm). The lowest velocities were observed for maximal rainfall (>106 mm) associated with the highest temperatures (>22.2°C). Overall, the effect of weather on the velocity of spread was higher at the one month-lag than at the two month-lag (Figure 3B).

**Figure 5:**
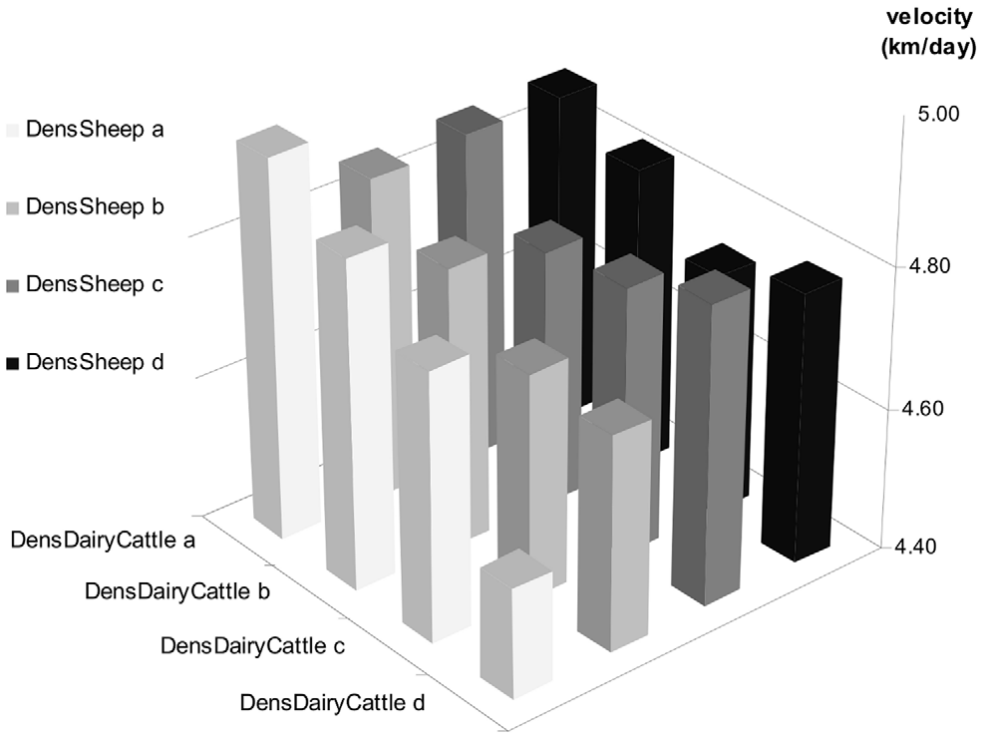
Effect of host availability on the velocity of BTV-8 spread in France in 2007–2008. The effect of the interaction between the densities of dairy cattle and sheep is represented through the value of the predicted velocity associated with each combination of cattle and sheep densities.

The effect of host availability variables is presented in Figure 5 and Table S5C. The velocity of BT spread was associated with dairy cattle and sheep densities, while the density of beef cattle had no effect. Moreover, the effect of dairy cattle density was modified by sheep density. The highest and lowest values of velocity were observed when sheep is absent, the highest velocities being observed for minimal density of dairy cattle (<4 dairy cattle/km^2^) and the lowest velocities with maximal density of dairy cattle (>40 animals/km^2^). Furthermore, for a given density of sheep, increasing the density of dairy cattle decreased the velocity of BT spread.

The level of immunity in cattle herds of a municipality, measured by the percentage of bovine vaccine doses acquired in the department 2 months prior to the first clinical case reported in the municipality, also influenced the velocity of BT spread. As expected, vaccination was associated negatively with the velocity of BT spread. In comparison to the velocity in municipalities where no vaccine was acquired in the department, the velocity decreased by 0.22 km/day when more than 30% of the vaccines were acquired in the department, and by 0.15 km/day when the percentage of vaccines acquired ranged between 1 and 30%, for an average velocity of 5 km/day across the country.

Regarding the five landscape-related variables, two variables had a larger influence on the velocity of BT spread (Figure 3B): the percentage of surface covered by pastures and by arable lands. Both were positively associated with the velocity of spread with a similar effect: a 10% increase in the percentage of pastures or arable land increased the velocity by 0.04 km/day.

**Figure 6:**
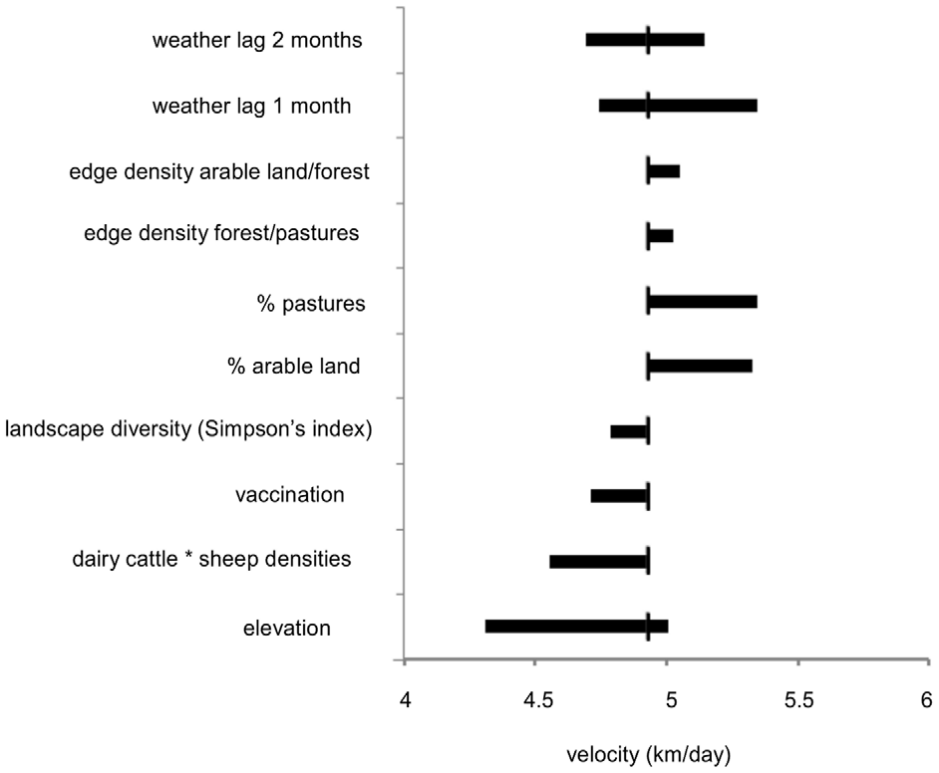
Empirical range of variations in velocities. Range of velocities obtained while changing each covariate across its observed range and holding all other covariates constant. The large horizontal bar represents the range of velocities obtained when the covariate varies between its maximal and minimal observed value, the average velocity of 4.9 km/day is represented by the small vertical stroke crossing the horizontal bar.

Finally, the range of velocities obtained while changing each covariate across its observed range and holding all other covariates constant is presented in Figure 6. The graph allows one to visualize if the observed values of a given covariate mainly induced a decrease or an increase in velocity compared to its average value of 5 km/day.

## Discussion

Several studies modeled the risk of BTV infection in relation to ecological correlates [21], [22], [25], [28], [60], but none investigated environmental factors facilitating the spread of the infection. While some studies investigated the impact of environmental factors on the diffusion of *Culicoides* [61]–[64], our work is the first attempt to model the velocity of BT spread while accounting for ecological variables related to *i*) vector abundance and activity (meteorological-related variables, elevation), *ii*) host-availability, and *iii*) landscape-related variables. We found that ecological variables related to *Culicoides* vectors (elevation, temperature and rainfall) were the main factors influencing the velocity of BT spread, although both host-availability and landscape-related variables also played a role.

Geography and climate have a strong influence on the spread and transmission of BTV because the distribution, abundance, activity and competence of adult vectors are influenced by weather [45], [65]. Indeed, many *Culicoides* life cycle parameters are related strongly to meteorological conditions: temperature, humidity, wind speed [47], [65]–[67]. Temperature is particularly important as it influences the recruitment, development, activity, survival and competence of *Culicoides* vectors [45]. Accordingly, temperature was identified as an important factor in determining the magnitude of the basic reproduction number R_0_ for BTV [18], [26], [27], [31]: BT transmission is optimal at a mean temperature of 20–25°C and decreases at warmer and cooler temperatures. Precipitation and daily maximum temperature also were the most useful climatic variables in predicting the BT infections status of cattle herds in Australia [67], [68], and Boyer et al. [69] found a positive association between the risk of BTV seropositivity and temperature in the United States. However, the effect of meteorological conditions on BT transmission rate is complex. In Australia, Ward [70] showed that the incidence rate of cattle herds depends on the total monthly rainfall recorded two months before the seroconversion of cattle as well as temperature recorded one month before seroconversion. Moreover, in addition to the crude abundance of *Culicoides*, their flying activity is important for disease transmission. Two studies reported the effect of meteorological conditions on flying activities [47], [71]. Broadly speaking, larger *Culicoides* catches were associated with higher temperatures, lower wind speeds and no rainfall at sunset. Heavy rainfall inhibited the flying activity of *Culicoides* populations. Overall, our findings are consistent with these studies. We observed the highest velocities of BT spread when the monthly average of maximum daily temperature at the one month-lag was between 21 and 26°C with minimal monthly rainfall. In contrast, the lowest velocities were observed for maximal rainfall associated with extreme temperature at the one month-lag and for maximal rainfall associated with maximal temperature at the two month-lag. Heavy rainfall thus decreased the velocity of spread of BTV while medium temperatures (around 20°C) increased it.

In addition to the effect of meteorological conditions, elevation was the most important ecological factors influencing the velocity of BT spread, with the lowest velocities observed at the highest elevation range. This may be due to the barrier effect associated with mountain areas, which may limit the dispersal of *Culicoides.* In Australia, speed of dispersal of *C. brevitarsis* is influenced by elevation with increasing altitude acting as barriers [61]. However, in contrast with *C. imicola*, which is never found at high altitudes in temperate regions, large populations of the Obsoletus Complex can be found in European areas above 1,000 m [42], [72]. The presence of vectors nevertheless does not imply viral activity. Indeed, both vector competence, *i.e.*, the ability of a vector to support virus infection, replication and dissemination, and vectorial capacity, *i.e.*, the ability of the vector population to transmit a pathogen, are subject to environmental modifications [73]. Obsoletus Complex midges caught in different regions of United Kingdom showed variations in susceptibility to BTV infection [74]. One may also expect that populations of vector located at higher altitudes possibly present lower competence and vectorial capacity because of lower temperatures, which would then decrease the velocity of spread of the infection at higher elevations. Further studies on the competence of the Obsoletus Complex are needed to elucidate the change in vector competence or vectorial capacity in relation to the geographical characteristics of the vector populations.

Interestingly, among the host-availability variables, the dairy cattle density had a large influence on the velocity of BT spread with the slowest velocities associated with the highest densities. Similarly, a negative relationship between the density of dairy cattle farms and BTV-4 occurrence was found in northern Spain [25]. The negative association between velocity of BT spread and dairy cattle density may be due to dairy cattle management practices. First, unlike sheep and beef cows, which may pasture far from the farm buildings with herds disseminated throughout landscape, dairy cows pasture close to buildings, creating localized clusters of hosts and more discontinuous host pattern availability. Because of the limited active dispersion of *Culicoides*, pattern of host regularly spaced within landscape is more favorable to BT diffusion than having locally clustered hosts, and it thus may explain the negative association of BT velocity with dairy cattle density. Second, after the milking process in the late afternoon, dairy cows often are kept indoors over-night to facilitate milking the following morning. Dairy cows thus may be less exposed to biting midges during times of maximum vector activity, *i.e.*, from dusk to sunrise [45]. In the Netherlands, Santman-Berends et al. [30] found a higher BTV-8 seroprevalence rate in cattle that grazed outdoors throughout the day and night compared to that in cattle pasturing only during the day. Similarly, Baylis et al. [75] showed that stabling could be useful to decrease BTV transmission by decreasing the risk of animals receiving bites from *C. obsoletus.*

Several other factors also affected the velocity of BT spread, but with a slighter effect. Regarding landscape-related variables, the percentage of surface covered by pastures or arable land as well as the edge density between forest and each of these two land cover were positively associated with the velocity of BT spread (Table 2). Furthermore, the percentage of pastures and arable land had a larger influence than edge density variables (Figure 3B and 6). The highest velocities of BT spread were observed for municipalities with the highest percentage of surface covered by pastures or arable land as well as for municipalities with the highest edge density between forest and pasture or forest and arable land. Overall these findings are consistent with previous results: high lengths of edges of woodland and open prairies were associated with high BT risk in Corsica [28], and edge densities between arable land and forests, and between pastures and forests, were identified as BTV-8 seropositivity risk factors for cattle in France [21]. As suggested by these authors, edges may provide meeting points between hosts (domestic and wild ruminants) and vectors (*Culicoides*). Pastures are occupied by domestic ruminants and also may serve as feeding areas for wildlife during the night. Forests provide breeding [42], [43] and resting [72] sites for Obsoletus Group as well as resting areas for wild ruminants. Finally, arable land may serve as feeding areas for wild ruminants [21]. Edges between these three habitats may thus facilitate contacts between BT vectors and hosts, while large surface covered by pastures or arable land may indicate large populations of domestic and wild hosts. Overall, these landscape characteristics may ultimately increase the rate of BT transmission. The only landscape-related variable that was negatively associated with the velocity is the Simpson's diversity index: the areas with higher diversity, *i.e.*, higher Simpson's diversity index, were associated with a lower velocity of spread. An increase of 0.1 in Simpson's diversity index decreased the velocity by 0.02 km/day. Similarly, Guis et al. [28] found a negative relationship between landscape diversity and the risk of BT due to *C. imicola* in Corsica.

Finally, we found that BT spread was slower in municipalities with a higher immunity. It is worth noting that despite the very crude proxy used to estimate the immunity of the cattle population (the percentage of vaccines acquired in a department two months prior to the date of the first clinical case in the municipality), the effect of vaccination was still identified. Further studies including more precise information on the vaccination rate would enable the amount by which vaccination decreases the velocity of BT spread to be quantified precisely.

Regarding the model performance in term of predictive power, the squared Pearson correlation coefficient and RMSE statistics were high for the training dataset, but relatively low for the validation dataset. Overall, this discrepancy may be taken as an indication of over-fitting. Indeed, the final model may capture too much of the specificity of the spatial dependencies and structure of the training dataset [76]. Hence, the differences between the two datasets have probably induced the poor predictive power of the model. It is consequently worth noting that the model is not suitable for making predictions for new data observations. It is well known that ignoring spatial autocorrelation can lead to overestimating environmental effects on species abundance [48], [59]. However, this issue still needs to be systematically addressed in epidemiological studies. In our study, we show how SAR_err_ models can be used to integrate autocorrelation into the analysis of environmental factors influencing the spread of an infectious disease. We demonstrated that using simple OLS and ignoring spatial autocorrelation would lead to overestimate the effect of strongly spatially structured environmental variables on disease spread. Accounting for spatial autocorrelation greatly improved the model fit, but also changed the conclusions regarding the relative importance of environmental factors on the velocity of BT spread. As previously shown in ecological studies on species diversity and distribution [77]–[79], such coefficient shifts underscore how the relative importance of environmental variables can be misconstrued when spatial autocorrelation is not controlled for. Accounting for spatial autocorrelation in epidemiological studies of disease spread is therefore critical to avoid misleading conclusions. We consequently strongly encourage researchers to routinely address the issue of spatial autocorrelation.

Finally, three potential weaknesses need to be considered. First, we did not account for wind-mediated vector movements on BT spread although it was found to help predict the pattern of BTV infections on a regional scale [22], [24]. The role of wind remains difficult to study because little is known about the conditions and characteristics of the wind events (such as height, temperature, humidity and speed) that can lead to midge transport, and because wind is highly heterogeneous in space and time hindering its modelling at fine scales. Second, as precise estimations of *Culicoides* abundance and activity were unavailable, we used environmental conditions known to affect biting midges to approximate both vector abundance and activity. Using field-collected entomological data to model vector abundance and activity from meteorological- and landscape-related variables would help confirm the role of the different environmental variables and their interpretation. Third, as mentioned above, a coarse proxy of vaccination was used as a measure of the level of host immunity. Overall, these shortcomings can explain that despite our use of spatial simultaneous autoregressive models, we failed to completely remove spatial autocorrelation in the residuals. Indeed, the residuals of the SAR_err_ model remained slightly autocorrelated. Residual autocorrelation in autoregressive models has been described previously [48], [77] and may be due to missing important variables. Failing to include or poorly measuring an important explanatory variable that in itself is highly autocorrelated thus may lead to autocorrelation of the residuals [1], [54], [79].

Despite these shortcomings the model presented a good explanatory power with a squared Pearson correlation coefficient of 0.93. Further work is still needed to improve our knowledge of the factors influencing BT spread. Specifically, future research into the distribution, activity and behavior of *Culicoides* is crucial. BT is a vector-borne disease and, as highlighted by our results, its spread is very much influenced by its *Culicoides* vectors [17].

## Conclusion

In this study we examined environmental factors influencing the velocity of BT spread and determined which ones were the most important drivers of disease progression. Our findings emphasize the importance of ecological factors associated with vector abundance and activity. Indeed, we found that elevation and meteorological conditions were the most important drivers of BT diffusion, as well as the density of dairy cattle, which was negatively correlated with the velocity of BT spread. These findings add substantially to our understanding of BT spread in a temperate climate. Importantly, the modelling approach used in this paper is general enough to be used easily to investigate the importance of environmental factors influencing the diffusion of other infectious diseases.

## Supporting Information

Text S1 **Description of the covariates related to host availability, vaccination and meteorological conditions.**
(PDF)

Text S2 **Description of the SAR_err_ models and choice of the weights style of the neighbourhood matrix.**
(PDF)

Table S1 **Land cover repartition of arable land, pastures, and deciduous and mixed forests in the 5,993 French municipalities.**
(PDF)

Table S2 **Lagrange Multiplier test.**
(PDF)

Table S3 **Number of parameters, Log likelihood, and AIC of the SAR_err_ models fitted to the 4,495 French municipalities.**
(PDF)

Table S4 **Parameter estimates, 95% Confidence Interval and p-values of the best Ordinary Least Square (OLS) model for the 4,495 municipalities.**
(PDF)

Table S5 **Velocity values estimated from the selected SAR_err_ model for each combination of A) rainfall and maximal temperature at the one month-lag, B) rainfall and maximal temperature at the two month-lag, and C) density of sheep and dairy cattle.**
(PDF)
