# Supplementary material for: Why Did Bluetongue Spread the Way It Did? Environmental Factors Influencing the Velocity of Bluetongue Virus Serotype 8 Epizootic Wave in France
Source: PLoS One. 2012 Aug 15;7(8):e43360. doi: 10.1371/journal.pone.0043360 (PMC3419712; doi:10.1371/journal.pone.0043360)
Supplement: Text S1 — Description of the covariates related to host availability, vaccination and meteorological conditions. (PDF) [file pone.0043360.s001.pdf]

Text S1. Description of the covariates related to host availability, vaccination and meteorological conditions

Host availability variables. Density of beef cattle and dairy cattle in September 2007 was obtained from the French National Cattle Register (BDNI). Because of maternal immunity, we only considered cattle over 2 months old to reflect the population size of susceptible hosts. Sheep density was obtained from the exhaustive census realized throughout France by the French Ministry of Agriculture in 2000, the most recent estimates we could obtain. Overall, the global number of sheep decreased between 2000 and 2007 in France. However, the relative differences of sheep density across geographical areas were similar between 2000 and 2007. We therefore assumed that the 2000 census approximately reflected the relative number of sheep per municipality in 2007. Densities were expressed in number of animals per km^2.

Vaccination. Precise data on BTV-8 vaccination were not available so we defined a proxy to estimate the relative immunity in the municipality when the first clinical case was detected. The vaccination data which was available was the number of BTV-8 bovine vaccine doses that each department (administrative unit grouping between 200 and 800 municipalities) had acquired prior to the detection of the first clinical case. This number varied because the doses were issued to departments in batches, and each department may not yet have acquired the total number required (corresponding to the number of cattle to be vaccinated in the department) when the first clinical case was declared on its territory. We consequently used the ratio between the number of vaccine doses acquired and the total number required in a department as a proxy of the relative immunity in a department. The bovine vaccine used was the Intervet Bovilis BTV-8®. Two injections three weeks apart are needed and immunity is acquired three weeks after the second injection. Hypothesizing that there was a delay of two to three weeks between the arrival of vaccine doses in a department and their use in the field by veterinarians, we used the percentage of vaccine doses acquired in a department two

months prior to the date of the first BTV-8 clinical case in a municipality as a proxy of the relative immunity in a municipality.

Meteorological-related variables. Both the BTV transmission cycle and the lifecycle of its *Culicoides* vectors are affected by temperature and moisture [11,30]. Short term meteorological conditions rapidly can change the age structure and density of *Culicoides* populations, thus modifying the number of midges that can transmit the virus, and consequently the rate of disease transmission [31]. Meteorological conditions also affect the daily flight activity of *Culicoides* vectors [32]. We thus were interested in capturing the meteorological conditions around the period at which the first animal became infected in a municipality. The exact date of infection of the first animal in each municipality was unknown, but we used the earliest date of clinical signs. Given that the first clinical signs occur 6-16 days post-infection (PI) in sheep [30,33] and 6-8 days PI in cattle [34], we reasonably could assume a 10-day incubation period between the infectious bite and the detection of symptoms by the farmer. To account for the uncertainty of the date of the infectious bite, we investigated the effect of temperature and rainfall up to two months prior to the date of the first clinical case reported in each municipality. We used SAFRAN meteorological data between January 2007 and December 2008. SAFRAN is a meteorological analysis system provided by Météo-France, which performs a fine-scale atmospheric analysis using data from the Météo-France automatic meteorological network [26,35]. SAFRAN provided the 2-m air temperatures and rainfall data over an 8 x 8 km square grid. Total rainfall (in mm) and the average of maximum daily temperatures (in °C) were obtained over 10-day periods (between the 1st and 10th, the 11th and 20th, and the 21st and 30th of each month). The 10-day period during which the first clinical case of BTV-8 was reported in each municipality was attributed the letter, D. We then computed the average maximum daily temperatures and rainfall for the month before the first BTV-8 case by using the data from the

D-1, D-2 and D-3 10-day periods, which combined formed the one month-lag. Similarly, we used the D-4, D-5 and D-6 10-day periods to compute the monthly average of maximal daily temperatures and monthly total rainfall two months before the first case of BTV-8, *i.e.*, the two month-lag.
