# Supplementary material for: Why Did Bluetongue Spread the Way It Did? Environmental Factors Influencing the Velocity of Bluetongue Virus Serotype 8 Epizootic Wave in France
Source: PLoS One. 2012 Aug 15;7(8):e43360. doi: 10.1371/journal.pone.0043360 (PMC3419712; doi:10.1371/journal.pone.0043360)
Supplement: Text S2 — Description of the SARerr models and choice of the weights style of the neighbourhood matrix. (PDF) [file pone.0043360.s002.pdf]

Text S2. Description of the SAR_{err} models and choice of the weights style of the neighbourhood matrix

Based on the results of the Lagrange Multiplier tests (Table S2), we applied the spatial error form of the SAR model (hereafter referred to as spatial error model or SAR_{err} model). Briefly, the usual OLS regression model $Y = \beta X_i + \varepsilon$ is complemented by a term ($\lambda W\mu$), which represents the spatial structure (λW) in the spatially dependent error term (μ) [1]. The SAR_{err} model thus takes the form $Y = \beta X_i + \lambda W\mu + \varepsilon$ where λ is the spatial autoregression coefficient, W is the spatial weights matrix and μ the spatially dependent error term.

Both the criteria used to define neighbours and the choice of the weights styles of the neighbourhood matrix may affect the results of SAR models. It is thus important to choose them carefully based on biological considerations [2]. An appropriate neighbourhood size is the maximum distance at which the residuals from an OLS model are autocorrelated [3]. This distance was judged from the semi-variogram of the OLS residuals (Figure 2). Residuals were autocorrelated until 200 km so we considered a 200 km radius spatial neighbourhood. This means that weights are non-zero when two locations are within 200 km of each other.

The choice of the weights style of the neighbourhood matrix may affect the results of SAR models, so we compared two criteria and three weights styles for the neighbourhood matrix in preliminary analyses. Regarding the two different neighbour weight definitions, we firstly set the weights to be proportional to the inverse distance between the centroids of the municipality (noted 1/d in the following table), which is the neighbour weight criteria used whether the strength of the neighbour relationships attenuates with distance. Second, we also used the default and classic neighbour weight criteria, which assumes no inverse relationships between the weights and the distance, (noted nb in the following table).

Numerous methods are available for coding the spatial weights matrix [2,4]. The most frequently used are (1) the binary coding 'B' for which locations are either listed as neighbours or not; (2) the row-standardisation 'W' which scales the covariances based on the number of neighbours of each row of the spatial weights matrix; and (3) the variance stabilization 'S' which stabilizes variances by summing over all links. We tested these three coding styles with the two above mentioned neighbour weight criteria.

All of the SAR_{err} models were run in a preliminary step with the full model (containing all the candidate covariates and the four interactions). We selected an appropriate weight function based on AIC. The model with the lowest AIC is the model using a neighbourhood matrix with an inverse distance criteria and a style 'W'. However, for the style 'W' the weights vary between the unity divided by the largest and smallest numbers of neighbours. Consequently, the weights for links originating at areas with few neighbours are larger than those originating at areas with many neighbours, which would bias the analysis by giving more weight to entities on the edge of the study area. Instead, the variance-stabilising coding scheme 'S' moderates this effect. Consequently, we selected the model with the 'S' coding style and the inverse distance criteria (1/d) which had the second lowest AIC. From an epidemiological point of view this means that the influence of neighbouring municipalities are decreasing with increasing distances and that municipalities at the edge of the study area or with few neighbours are given less weight. Hence, for a given municipality the residual of the model, *i.e*, the difference between the observed and predicted velocity, is correlated with the residuals of the neighbouring municipalities located within a 200 km circle around it. Moreover, the correlation between the residuals is decreasing with increasing distances, and the residuals of the municipalities located at the edge of the study area or with few neighbours are given less weight in influencing the residuals of the considered municipality.

Table: AIC of the six full SAR_{err} models tested for the 4,495 French municipalities. The selected model is in bold.

neighbour weight criteria	coding style	AIC
1/d	B	8308
1/d	W	5278
**1/d**	**S**	**6982**
nb	B	9192
nb	W	8845
nb	S	8472

Reference

1. Dormann CF, McPherson JM, Araújo MB, Bivand R, Bolliger J, et al. (2007) Methods to account for spatial autocorrelation in the analysis of species distributional data: a review. Ecography 30: 609-628.
2. Bivand RS, Pebesma EJ, Gómez-Rubio V (2008) Applied spatial data analysis with R.; Gentleman R, Hornik K, Parmigiani G, editors. New-York: Springer. 378 p.
3. Lichstein JW, Simons TR, Shriner SA, Franzreb KE (2002) Spatial autocorrelation and autoregressive models in ecology. Ecological Monographs 72: 445-463.
4. R package version 0.5-37 (2011) Package spdep: spatial dependence: weighting schemes, statistics, and models
