# Supplementary material for: Why Did Bluetongue Spread the Way It Did? Environmental Factors Influencing the Velocity of Bluetongue Virus Serotype 8 Epizootic Wave in France
Source: PLoS One. 2012 Aug 15;7(8):e43360. doi: 10.1371/journal.pone.0043360 (PMC3419712; doi:10.1371/journal.pone.0043360)
Supplement: Table S1 — Land cover repartition of arable land, pastures, and deciduous and mixed forests in the 5,993 French municipalities. (PDF) [file pone.0043360.s003.pdf]

Supplementary Table 1. Land cover repartition of arable land, pastures, and deciduous and mixed forests in the 5,993 French municipalities.

Identification code for land cover classes	Land cover classes	CLC classes	Number of municipalities with null values (%)
arable	Arable land	211-213	1245 (21)
pasture	Pastures	231	417 (7)
forest	Deciduous and mixed forests	311, 313, 324	713 (12)

Source: Coordination de l'Information sur l'Environnement (CORINE) land cover database, 2006 version. Each CLC class is identified by three numbers.
