# Supplementary material for: Why Did Bluetongue Spread the Way It Did? Environmental Factors Influencing the Velocity of Bluetongue Virus Serotype 8 Epizootic Wave in France
Source: PLoS One. 2012 Aug 15;7(8):e43360. doi: 10.1371/journal.pone.0043360 (PMC3419712; doi:10.1371/journal.pone.0043360)
Supplement: Table S2 — Lagrange Multiplier test. (PDF) [file pone.0043360.s004.pdf]

Supplementary Table 2. Lagrange Multiplier test.

test	statistics	p-value
LMerr	102224	<0.001
LMlag	3669	<0.001
RLMerr	99980	<0.001
RLMlag	1425	<0.001
