# Supplementary material for: Why Did Bluetongue Spread the Way It Did? Environmental Factors Influencing the Velocity of Bluetongue Virus Serotype 8 Epizootic Wave in France
Source: PLoS One. 2012 Aug 15;7(8):e43360. doi: 10.1371/journal.pone.0043360 (PMC3419712; doi:10.1371/journal.pone.0043360)
Supplement: Table S3 — Number of parameters, Log likelihood, and AIC of the SARerr models fitted to the 4,495 French municipalities. (PDF) [file pone.0043360.s005.pdf]

Supplementary Table 3. Number of parameters (np), Log likelihood, and AIC of the SAR_{err} models for the 4,495 municipalities. We used a hierarchical approach to achieve a greater discrimination between covariates (see Materials and Methods for details). The selected model within each set of models is highlighted in grey. The selected model after grouping the remaining covariates and performing model selection is in bold. See Table 1 for descriptions of covariates.

model	np	Log likelihood	AIC
**Models with landscape covariates**			
elevation + SIDI + p_arable + p_pasture + p_forest +forest-pasture + arable-forest + arable-pasture	19	-3703.3	7444.6
without arable-pasture	16	-3706.2	7444.4
without arable-pasture and arable-forest	13	-3711.5	7449.1
without arable-pasture and forest-pasture	13	-3710.8	7447.5
without arable-pasture and p_forest	15	-3707.1	7444.2
without arable-pasture, p_forest and p_pasture	14	-3724.6	7477.2
without arable-pasture, p_forest and p_arable	14	-3719.6	7467.2
without arable-pasture, p_forest and SIDI	14	-3713.9	7455.8
without arable-pasture, p_forest and elevation	12	-3860.3	7744.6
without arable-pasture, p_forest and forest-pasture	12	-3715.0	7454.0
**Models with host availability covariates**			
DensSheep*DensBeef_Cattle + DensSheep*DensDairy_Cattle + vaccination	32	-3826.4	7716.7
without DensSheep*DensBeef_Cattle	23	-3836.7	7719.4
without DensSheep*DensDairy_Cattle	23	-3861.5	7769.0
without vaccination	30	-3860.4	7780.9
without DensSheep	11	-3873.7	7769.4
without DensBeef_Cattle	20	-3841.1	7722.2
without DensDairy_Cattle	20	-3899.5	7839.0

**Models with meteorological covariates**			
Rain_lag1*Tmax_lag1 + Rain_lag2*Tmax_lag2	33	-3772.1	7610.3
without Rain_lag1*Tmax_lag1	24	-3808.2	7664.4
without Rain_lag2*Tmax_lag2	24	-3809.2	7666.3
without Rain_lag1	21	-3833.8	7709.7
without Tmax_lag1	21	-3849.4	7740.8
without Rain_lag2	21	-3818.7	7679.5
without Tmax_lag2	21	-3843.7	7729.4
**Models grouping the remaining landscape, host availability and meteorological covariates**			
elevation + SIDI + p_arable + p_pasture +forest-pasture + arable-forest + DensSheep*DensBeef_Cattle + DensSheep*DensDairy_Cattle + vaccination + Rain_lag1*Tmax_lag1 + Rain_lag2*Tmax_lag2	74	-3428.2	7004.4
without elevation	73	-3448.9	7043.8
without SIDI	73	-3431.0	7008.0
without p_arable	73	-3448.9	7043.8
without p_pasture	73	-3446.5	7038.9
without arable-forest	71	-3436.5	7015.0
without forest-pasture	71	-3432.1	7006.1
without the interaction between DensSheep and DensDairy_Cattle	65	-3450.6	7031.3
without the interaction between DensSheep and DensBeef_Cattle	65	-3437.5	7005.1
without DensDairy_Cattle	62	-3496.6	7117.2
**without DensBeef_Cattle**	**62**	**-3440.9**	**7005.8**
without DensSheep	53	-3460.6	7027.2
without vaccination	72	-3456.9	7057.7
without the interaction between Rain_lag1*Tmax_lag1	65	-3483.2	7069.5
without the interaction between Rain_lag2*Tmax_lag2	65	-3465.9	7061.9
without Rain_lag1	62	-3496.1	7116.3
without Tmax_lag1	62	-3507.1	7138.3
without Rain_lag2	62	-3472.5	7069.0
without Tmax_lag2	62	-3497.7	7119.3
without forest-pasture and DensBeef_Cattle	59	-3444.8	7007.6
