# Supplementary material for: Why Did Bluetongue Spread the Way It Did? Environmental Factors Influencing the Velocity of Bluetongue Virus Serotype 8 Epizootic Wave in France
Source: PLoS One. 2012 Aug 15;7(8):e43360. doi: 10.1371/journal.pone.0043360 (PMC3419712; doi:10.1371/journal.pone.0043360)
Supplement: Table S4 — Parameter estimates, 95% Confidence Interval and p-values of the best Ordinary Least Square (OLS) model for the 4,495 municipalities. (PDF) [file pone.0043360.s006.pdf]

Supplementary Table 4. Parameter estimates, 95% Confidence Interval (CI) and p-values of the best Ordinary Least Square (OLS) model for the 4498 municipalities.

covariates	classe	coefficient	95%CI	p-value
intercept		5.93	5.584 ; 6.277	<0.001
elevation	b	0.04	-0.046 ; 0.121	0.38
	c	-0.93	-1.035 ; -0.828	<0.001
	d	-1.54	-1.660 ; -1.414	<0.001
DensBeef_Cattle	b	0.19	0.071 ; 0.313	<0.01
	c	0.12	0.001 ; 0.249	0.05
	d	0.10	-0.026 ; 0.226	0.12
DensDairy_Cattle	b	-0.24	-0.349 ; -0.138	<0.001
	c	-0.49	-0.606 ; -0.373	<0.001
	d	-1.04	-1.155 ; -0.926	<0.001
DensSheep	b	-0.07	-0.285 ; 0.152	0.55
	c	0.17	-0.048 ; 0.392	0.13
	d	0.24	0.024 ; 0.458	0.03
vaccination	b	-0.37	-0.458 ; -0.280	<0.001
	c	-0.60	-0.691 ; -0.512	<0.001
Rain_lag1	b	0.07	-0.088 ; 0.229	0.38
	c	0.01	-0.163 ; 0.177	0.94
	d	-0.13	-0.302 ; 0.034	0.12
Tmax_lag1	b	0.01	-0.169 ; 0.187	0.92
	c	0.22	0.046 ; 0.396	0.01
	d	0.39	0.213 ; 0.562	<0.001
Rain_lag2	b	0.25	0.073 ; 0.429	0.01
	c	0.31	0.122 ; 0.496	<0.01
	d	0.57	0.403 ; 0.741	<0.001
Tmax_lag2	b	-0.28	-0.463 ; -0.102	<0.01
	c	-0.26	-0.454 ; -0.075	0.01
	d	0.44	0.261 ; 0.620	<0.001
SIDI		-0.98	-1.299 ; -0.661	<0.001
p_arable		0.01	0.008 ; 0.014	<0.001
p_pasture		0.01	0.004 ; 0.010	<0.001
p_forest		0.01	0.003 ; 0.009	<0.001
arable-pasture	b	0.07	-0.025 ; 0.155	0.15
	c	-0.07	-0.185 ; 0.037	0.19
	d	-0.14	-0.274 ; -0.007	0.04
arable-forest	b	0.01	-0.079 ; 0.096	0.85
	c	0.04	-0.049 ; 0.138	0.35
	d	0.15	0.048 ; 0.261	0.01
forest-pasture	b	0.04	-0.052 ; 0.131	0.40
	c	0.17	0.061 ; 0.269	<0.01
	d	0.31	0.183 ; 0.434	<0.001
interactions				
1st term	2nd term			
DensBeef_Cattle b	DensSheep b	0.06	-0.178 ; 0.290	0.64
	DensSheep c	-0.14	-0.400 ; 0.118	0.29

	DensSheep d	-0.20	-0.466 ; 0.068	0.15
DensBeef_Cattle c	DensSheep b	0.17	-0.070 ; 0.416	0.16
	DensSheep c	-0.03	-0.291 ; 0.232	0.83
	DensSheep d	-0.03	-0.302 ; 0.245	0.84
DensBeef_Cattle d	DensSheep b	0.34	0.099 ; 0.590	0.01
	DensSheep c	0.39	0.137 ; 0.635	<0.01
	DensSheep d	0.47	0.228 ; 0.704	<0.001
DensDairy_Cattle b	DensSheep b	0.13	-0.078 ; 0.347	0.21
	DensSheep c	-0.03	-0.243 ; 0.185	0.79
	DensSheep d	0.09	-0.117 ; 0.294	0.40
DensDairy_Cattle c	DensSheep b	0.14	-0.099 ; 0.371	0.26
	DensSheep c	0.19	-0.054 ; 0.428	0.13
	DensSheep d	0.01	-0.237 ; 0.263	0.92
DensDairy_Cattle d	DensSheep b	0.56	0.331 ; 0.791	<0.001
	DensSheep c	0.86	0.628 ; 1.090	<0.001
	DensSheep d	0.49	0.221 ; 0.762	<0.001
Tmax_lag1 b	Rain_lag1 b	-0.11	-0.350 ; 0.127	0.36
	Rain_lag1 c	-0.04	-0.281 ; 0.197	0.73
	Rain_lag1 d	0.76	0.521 ; 0.998	<0.001
Tmax_lag1 c	Rain_lag1 b	-0.27	-0.494 ; -0.045	0.02
	Rain_lag1 c	-0.16	-0.391 ; 0.079	0.19
	Rain_lag1 d	-0.39	-0.633 ; -0.139	<0.01
Tmax_lag1 d	Rain_lag1 b	-0.30	-0.529 ; -0.064	0.01
	Rain_lag1 c	-0.61	-0.848 ; -0.378	<0.001
	Rain_lag1 d	-1.02	-1.249 ; -0.788	<0.001
Tmax_lag2 b	Rain_lag2 b	0.55	0.307 ; 0.791	<0.001
	Rain_lag2 c	0.62	0.377 ; 0.870	<0.001
	Rain_lag2 d	0.26	0.024 ; 0.498	0.03
Tmax_lag2 c	Rain_lag2 b	0.78	0.529 ; 1.034	<0.001
	Rain_lag2 c	0.52	0.266 ; 0.765	<0.001
	Rain_lag2 d	-0.75	-1.001 ; -0.491	<0.001
Tmax_lag2 d	Rain_lag2 b	-0.45	-0.673 ; -0.219	<0.001
	Rain_lag2 c	-0.95	-1.200 ; -0.709	<0.001
	Rain_lag2 d	-1.26	-1.517 ; -1.001	<0.001

See Table 1 for descriptions of covariates.
