# Supplementary material for: Why Did Bluetongue Spread the Way It Did? Environmental Factors Influencing the Velocity of Bluetongue Virus Serotype 8 Epizootic Wave in France
Source: PLoS One. 2012 Aug 15;7(8):e43360. doi: 10.1371/journal.pone.0043360 (PMC3419712; doi:10.1371/journal.pone.0043360)
Supplement: Table S5 — Velocity values estimated from the selected SARerr model for each combination of A) rainfall and maximal temperature at the one month-lag, B) rainfall and maximal temperature at the two month-lag, and C) density of sheep and dairy cattle. (PDF) [file pone.0043360.s007.pdf]

Supplementary Table 5. Velocity values estimated from the selected SAR_{err} model for each combination of A) rainfall and maximal temperature at the one month-lag, B) rainfall and maximal temperature at the two month-lag, and C) density of sheep and dairy cattle. Velocity values are reported holding all the other covariates constant, and for an average velocity of BT spread across the country of 4.93 km/day (Table 2). a, b, c, d represent classes with increasing values. See Table 1 for details on the range value of each class. In each table, the minimal and maximal velocities are highlighted in grey.

A

Lag 1		rain			
		a	b	c	d
Tmax	a	4.93	4.98	4.89	4.74
	b	5.01	4.99	5.05	5.21
	c	5.34	5.16	5.13	5.02
	d	5.28	5.13	5.08	4.86

B

Lag 2		rain			
		a	b	c	d
Tmax	a	4.93	4.93	5.09	5.05
	b	4.85	5.02	5.14	5.05
	c	4.93	5.09	5.00	4.79
	d	4.94	4.86	4.74	4.69

C

		DensDairy_Cattle			
		a	b	c	d
DensSheep	a	4.93	4.86	4.78	4.56
	b	4.85	4.79	4.72	4.70
	c	4.86	4.76	4.78	4.82
	d	4.87	4.83	4.74	4.78
